# Photoablation at single cell resolution and its application in the *Drosophila* epidermis and peripheral nervous system

**DOI:** 10.3389/fphys.2022.1093303

**Published:** 2023-01-04

**Authors:** Federica Mangione, Rocco D’Antuono, Nicolas Tapon

**Affiliations:** ^1^ Apoptosis and Proliferation Control Lab, The Francis Crick Institute, London, United Kingdom; ^2^ Crick Advanced Light Microscopy STP, The Francis Crick Institute, London, United Kingdom

**Keywords:** photoablation, epidermis, tactile bristle, peripheral nervous system, *Drosophila*

## Abstract

Tissues contain diverse cell populations that, together, make up physiologically functional units. A remarkable example is the animal epidermis, where neuronal and non-neuronal cells intermingle to allow somatosensory perception. In the peripheral nervous system (PNS), the tight association between heterogenous cell types poses challenges when the structural and physiological contributions of neuronal and surrounding cells need to be dissected with suitable precision. When genetic tools for cell-specific, spatiotemporally controlled gene expression are not available, targeted cell ablation represents a considerable obstacle. Here, we describe an efficient method to overcome this limitation and demonstrate its application to the study of the differentiating *Drosophila* epidermis and PNS. This methodology relies on the use of near infrared (NIR) femtosecond (fs) laser pulses for ablation of the desired cells at the desired time. We show how to confine the photodamage to the targeted cell to induce its death, without harming neighbouring tissues or structures. We validated our approach in the *Drosophila* PNS by studying the responses of photo-ablated neurons, non-neuronal cells, and the surrounding epidermis. Diverse cellular behaviours including cell extrusion, cell rearrangements and cell shape changes can be monitored *in vivo* immediately after damage, as well as for several hours post-ablation with high optical resolution using confocal microscopy. This methodology provides a flexible tool to ablate individual cells with high precision and study morphological responses to cell loss in targeted areas or neighbouring structures. We anticipate that this protocol can be easily adapted to other model systems and tissues.

## Introduction

**FIGURE 1:**
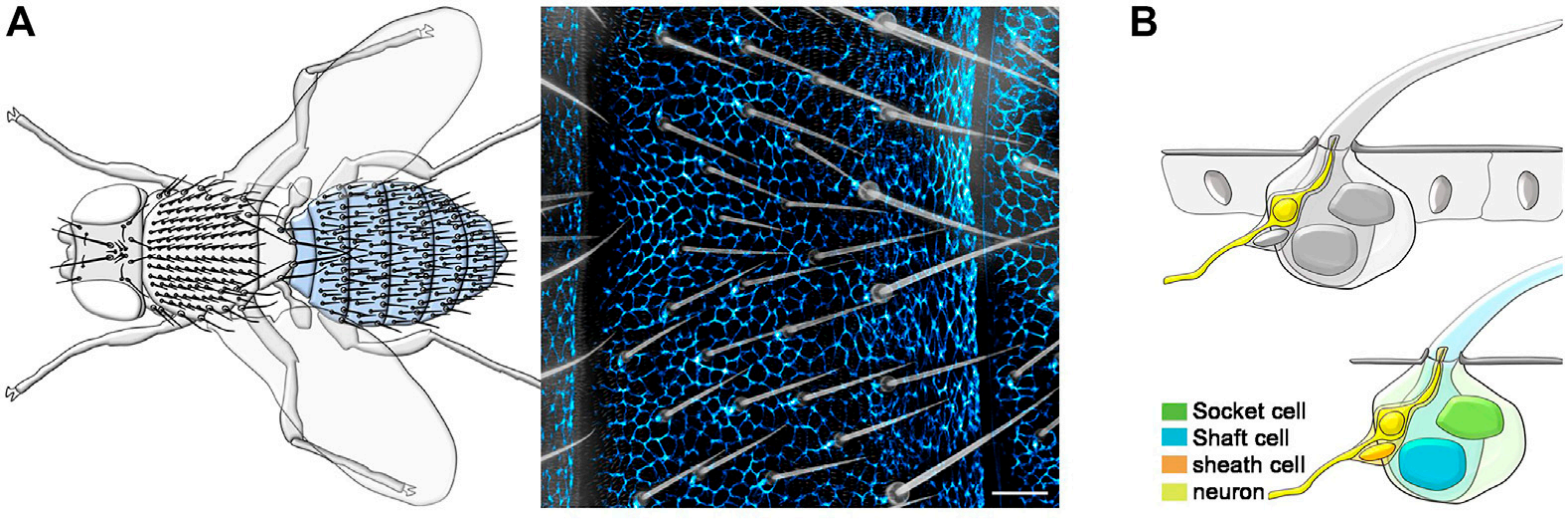
Overview of the adult *Drosophila* epidermis and PNS. **(A)** Left: Diagram showing the tactile bristles covering the dorsal epidermis of the adult fly. The adult abdominal epidermis is highlighted in light blue. Right: Image of the epidermal cuticle and associated tactile bristles of the adult abdomen. Epidermal cells are outlined by the expression of the junctional marker *Atp-α::GFP* (blue) and tactile bristles are visualized using bright field imaging. **(B)** Diagrams showing the tactile bristle and surrounding epidermal cells (Top) and the four cells of each tactile bristle (Bottom). Scale bars: 20 µm.

The ability to detect and respond to external stimuli is an essential feature of living organisms. In animals, most neurons of the peripheral nervous system (PNS) innervate specialized sensory organs to detect and convey these stimuli to the brain (Sulston and White, 1980; Lumpkin et al., 2010; Handler and Ginty, 2021). The adult *Drosophila melanogaster* PNS comprises thousands of sensory organs located in stereotyped positions throughout the body. The most abundant sensory organs are mechanosensory bristles (Held, 1991; Liu and Zhang, 2022), tactile hairs covering most of the adult epidermis (Figure 1A). All tactile bristle cells originate from the asymmetric cell division of a single precursor cell, the sensory organ precursor (SOP), which itself is selected by lateral inhibition within the epidermal layer (Gho et al., 1996; Lai and Orgogozo, 2004). Over the course of pupal development, each tactile bristle is subsequently embedded in the epidermis, where it encapsulates the sensory neuron (Figure 1B). Each developing tactile bristle comprises a pair of subepidermal cells, the sensory neuron and associated sheath glia, and a pair of cells that make up the exteroceptor structure (Hartenstein and Posakony, 1989), the Socket cell and the hair Shaft cell (Figure 1B). While the early development of the tactile bristles has been studied in detail, little is still known about the terminal differentiation of the constituent cells and supporting epidermis.

The tight association between sensory neurons, bristle cells and supporting epidermis makes genetic access to individual cell types particularly challenging. However, a deeper understanding of the structural and physiological contributions of each of these cell types to somatosensory perception would benefit from experimentally interfering with one cell at a time.

Targeted cell ablation is a powerful approach for studying cell type-specific functions during tissue morphogenesis, cell differentiation, or tissue regeneration *in vivo* (Liu et al., 2019). The ability to ablate cells is often limited by the genetic tools and accessibility of the target tissue. Currently, genetic tools for cell-specific, spatiotemporally controlled gene expression in differentiating bristles and PNS neurons targeting the adult epidermis are limited. To overcome this limitation, laser ablation constitutes a powerful optical tool. Classical studies in *C. elegans* using dye lasers and Nomarski optics demonstrated the power of laser ablations in probing the function of individual cells (Liu et al., 2019). More recently, laser ablation has successfully been used to create cellular or tissue lesions (Colombelli et al., 2007; Rauzi et al., 2015), probing mechanical properties (Rauzi et al., 2008; Shivakumar and Lenne, 2016; Davis et al., 2022) or dissecting the contribution of subcellular structures during morphogenesis (Collinet et al., 2015; Lopez-Gay et al., 2020).

Continuous or pulsed laser sources in the ultra-violet (UV) or near infrared (NIR) range have been used for tissue and cell ablation in various biological contexts (Colombelli et al., 2007; Fang-Yen et al., 2012; Pineda et al., 2015). NIR femtoseconds (fs) pulsed lasers, including those used for multiphoton microscopy (Denk et al., 1990; Denk, 1996), are particularly well suited for laser ablations experiments in cells and tissues. With NIR-fs pulsed lasers, non-linear absorption of NIR photons is spatially confined in a small focal volume, allowing precise cellular targeting at submicron resolution (Vogel et al., 2005). When local excitation through nonlinear absorption is induced by high power NIR-fs pulsed energy, regions within and around the target site are subjected to thermo-mechanical damage, a phenomenon called optical breakdown (Vogel et al., 2005; Tsai et al., 2009). We used this knowledge to implement an efficient method for photoablation of single cells using high power NIR-fs pulsed laser, while limiting thermo-mechanical damage to the target site.

Here, we describe a simple methodology to perform photoablation at single cell resolution and show its application in the study of the epidermis and sensory organs of the *Drosophila* PNS. Combining high-power NIR-fs pulsed laser technology with confocal microscopy, this protocol allows the ablation of individual cells with precise spatiotemporal control, followed by monitoring cell and tissue dynamics of adjacent cells. Using this methodology, we show that photodamage is confined to the targeted cell, while neighbouring tissues or structures are not affected. We anticipate that the settings and tuneable parameters described here can easily be adapted for successful single cell photoablation in diverse tissues and model organisms.

## Materials and Equipment

### Fly husbandry and stocks

Fly strains and crosses were raised on standard cornmeal food at 25°C and on a 12 h light/dark cycle. The following fly stocks were used in this study: *neur-GAL4* (Bellaiche et al., 2001), *elav-GAL4* (BDSC 458), *UAS-H2B-RFP* (Mayer et al., 2005), *UAS-mCD8::GFP* (BDSC 5130), UAS-GMA::GFP (BDSC 31776, referred to as *UAS-Actin::GFP*), *E-Cad::GFP* (Huang et al., 2009), *Atp-α::GFP* (DGGR 110860), *Ubi-RFP. nls* (BDSC 35496) and *Diap1-GFP* (Zhang et al., 2008).

### Sample preparation for live imaging and photoablation

Pupae were staged as described in (Bainbridge and Bownes, 1981) and timed employing puparium formation as a reference (hours After Puparium Formation—hAPF). Staged pupae were prepared for live imaging as described in (Mangione and Martin-Blanco, 2020). Briefly, under a dissection microscope, the pupal case was peeled off each pupa using forceps. Naked pupae were transferred to a glass-bottom dish, where each pupa was placed on a small drop of gas-permeable halocarbon oil to reduce refractive index mismatch during imaging with oil immersion objectives. Multiple pupae were mounted for each experiments using this procedure.

### Live imaging with confocal microscopy

In this study, we used inverted Laser Scanning Microscopes (LSM780 and LSM880, Carl Zeiss) equipped with high Numerical Aperture (NA) Objectives (40X/1.3 NA and 63X/1.4 NA oil immersion objective lens). Continuous wave (CW) solid state and Argon lasers were used for the imaging of samples containing GFP and RFP derivatives (laser lines 488 and 561 nm in microscope software, version ZEN 2.3 SP1 FP3 black, Carl Zeiss, Microscopy GmbH). The time interval and number of slices per *z*-stacks was adapted for each experiment, depending on the size and the developmental stage of the targeted cell. Laser power was kept to a minimum (*i.e*., typically below 20% for both the acquisition channels) to prevent photobleaching. Pupae were cultured to adulthood after imaging, showing unperturbed development.

### Photoablation of single cells

We used inverted Laser Scanning Microscopes (LSM780 or LSM880, Carl Zeiss Microscopy Ltd) equipped with Near Infrared (NIR) femtosecond (fs) pulsed laser (Titanium:Sapphire (Ti:Sa) laser; Chameleon Vision II, Coherent Inc), tunable from 680 to 1300 nm for multiphoton imaging. To perform photoablation experiments, the NIR-fs pulsed laser power, pixel dwell time, number of iterations, bleaching ROI size and focus *z*-position must be precisely controlled. All these parameters were tuned under the ZEN software (ZEN 2.3 SP1 FP3 black, Carl Zeiss Microscopy GmbH, Zeiss, with Bleaching tool tab activated) for each experiment, to ensure successful elimination of the targeted cells. For most of our experiments the MP laser was tuned at 780 nm and set at 70% power, with a pixel dwell time between 1 and 2 μs. Between 1 and 3 iterations were used to ablate circular ROIs ranging between 25 and 35 pixels in diameter (about 1.4 and 2.4 μm). The range of the z-stack was defined in relation to the centre of the stack, to which the NIR-fs pulses were directed (using the z-stack tool tab in centre mode). Following each laser ablation, each *z*-stack was then acquired in confocal mode to visualize cell behaviours shortly after photoablation.

### Image visualization and image analysis

All images and time-lapses were visualized and processed using Fiji (Schindelin et al., 2012). The Maximum Intensity Projection (Image > Stacks > Z-project) was used to display each *z*-stack in 2D. The Minimum Intensity Projection (Image > Stacks > Z-project) was used to display bright-field images of the epidermal cuticle. Some *z*-stacks were displayed in 3D using the 3D viewer option (Image > Stacks > 3D-project) or the Re-slice tool (Image > Stack > Re-slice) of Fiji. Mean fluorescence intensity before and after photobleaching experiments was measured with Fiji (Analyze > Measure > Mean Gray Value). Kymographs from time-lapses were made in Fiji with the Re-slice tool. The NumPy (Harris et al., 2020) package for Python was used for simulating the NIR-fs laser excitation profile, from a Gaussian distribution in the x axis and a Lorentzian distribution for the z axis.

## Method description

### Genetic toolkit for single cell photoablation

An essential prerequisite to successfully perform photoablation is to label target tissues or structures by the expression of fluorescent markers. Identifying the cell of interest and make its surrounding visible is indeed crucial for precise cellular targeting for photoablation. In this protocol, we use live imaging, so all image acquisition and analysis will depend on genetically encoded fluorescently labelled proteins. When possible, we recommend combining GFP and RFP reporter lines in the same experiments. For example, expression of membrane-localized GFP reporters together with the expression of an RFP-tagged nuclear marker allows the simultaneous visualization of both the geometry and nuclear position of each cell. This combination of cellular markers helps distinguish each cell from its neighbours within the epidermis and associated sensory organs. We also recommend the use of the GAL4/UAS (Brand and Perrimon, 1993) or similar binary systems (Lai and Lee, 2006; Potter et al., 2010; del Valle Rodriguez et al., 2011), to drive the expression of GFP/RFP reporter lines in discrete cellular subsets. For example, the use of the bristle-specific GAL4 driver *neuralized* (*neur-GAL4;* see Materials and Equipment) with membrane-localized and/or nuclear fluorescent reporters allows the identification of all bristle cells.

The desired pupal stage must be also selected before photoablation. One of the advantages of the pupa is its immobility, which makes epidermal development and differentiation accessible to live-imaging for over 3 days (Mangione and Martin-Blanco, 2018). Pre-pupal to pupal transitions (i.e*.,* between about 8-to-12 hAPF) and pupal to adult eclosion (i.e., by about 90 hAPF) must be avoided as major morphogenetic movements would hinder imaging. We recommend preparing multiple pupae for each experiment (Mangione and Martin-Blanco, 2020 and Materials and Equipment) so that photoablation experiments can be repeated various times and in multiple animals in each imaging session.

### Optical toolkit for single cell photoablation

**FIGURE 2:**
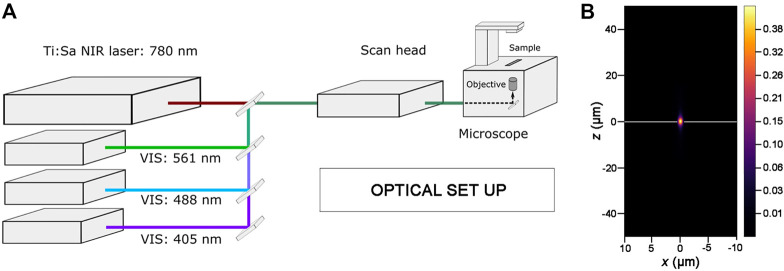
Optical set up for photoablation experiments. **(A)** Simplified diagram showing the key components of the optical set up used for photoablation. An inverted Laser Scanning Microscope, where the laser lines of the visible (VIS) range are highlighted, is equipped with a Ti:Sapphire Near Infrared femtosecond pulsed laser (Ti:Sa NIR-fs Laser; tunable from 680 nm to 1300 nm for multiphoton imaging). All laser lines are coupled to the electronic and optical components of the microscope (scan head) and can be tuned individually with the microscope software. The 488 nm and 561 nm laser lines are used for imaging GFP and RFP while the Ti:Sa NIR-fs, tuned at 780 nm, is used to get spatially confined photoablation. **(B)** Excitation/Photoablation efficiency profile for a Ti:Sa NIR-fs laser in the *xz* plane. The colour shows signal density ranging from 0 (low) to 0.43 (high) in arbitrary units (a.u.). Note that the higher density of the NIR-fs pulsed laser is confined to the focal plane (white line).

To perform photoablation and live-imaging, we use an inverted laser scanning confocal microscope equipped with a tunable NIR-fs pulsed laser source (Figure 2A; see also Materials and Equipment). Activation of each laser line (i.e., VIS laser module and NIR-fs pulsed laser line), attenuation of laser power for each line, and other acquisition parameters are managed within the microscope software (see Materials and Equipment). For laser scanning, we select the frame mode, and used a 1024 × 1024 pixels image frame size. We recommend setting the scan direction as bi-directional to reduce the scan time (i.e., the duration of the acquisition of the entire frame) during live imaging. For photoablation with the NIR-fs laser, both the dimension and position of the region of interest (ROI) to be ablated within the z-stack are defined using the microscope software (see Materials and Equipment). The number of iterations (the number of scans which are performed for bleaching the selected ROI in each experiment) and scan speed need to be optimized before starting the photoablation (see below). Decreasing the scan speed results in a longer pixel dwell (average time during which the laser beam is focused on each pixel), that can increase the efficiency of bleaching.

NIR-fs lasers emit pulsed radiations that allow the absorption of two or, in principle, more photons in a single quantum event (Denk, 1996; Rubart, 2004). The simultaneous absorption of the two photons induces an electronic transition in the fluorophore from the ground to the excited energy state, equivalent to the fluorescent excitation obtained in single photon mode by using laser lines in the visible range (Helmchen and Denk, 2005). The probability of absorption of multiple photons is non-zero only in a small volume centred in the focal plane where fluorescence excitation takes place. Therefore, the spatial profile of the NIR-fs laser beam is key parameter for predicting targeting precision for photoablation. As the optical resolution of a NIR-fs laser substantially improves in combination with high NA objectives (Rubart, 2004; Vogel et al., 2005), we used 1.3 NA or 1.4 NA objectives in all our experiments. As shown in Figure 2B, the spatial profile of the NIR-fs beam 780–800 nm with 1.3 NA objective shows minimal out-of-focus excitation, an essential requisite for performing highly localized photomanipulations.

### Single cell photoablation

Our workflow for photoablation at single cell resolution encompasses a few important steps that ensure successful elimination of the cell of interest, which are described below.

**FIGURE 3:**
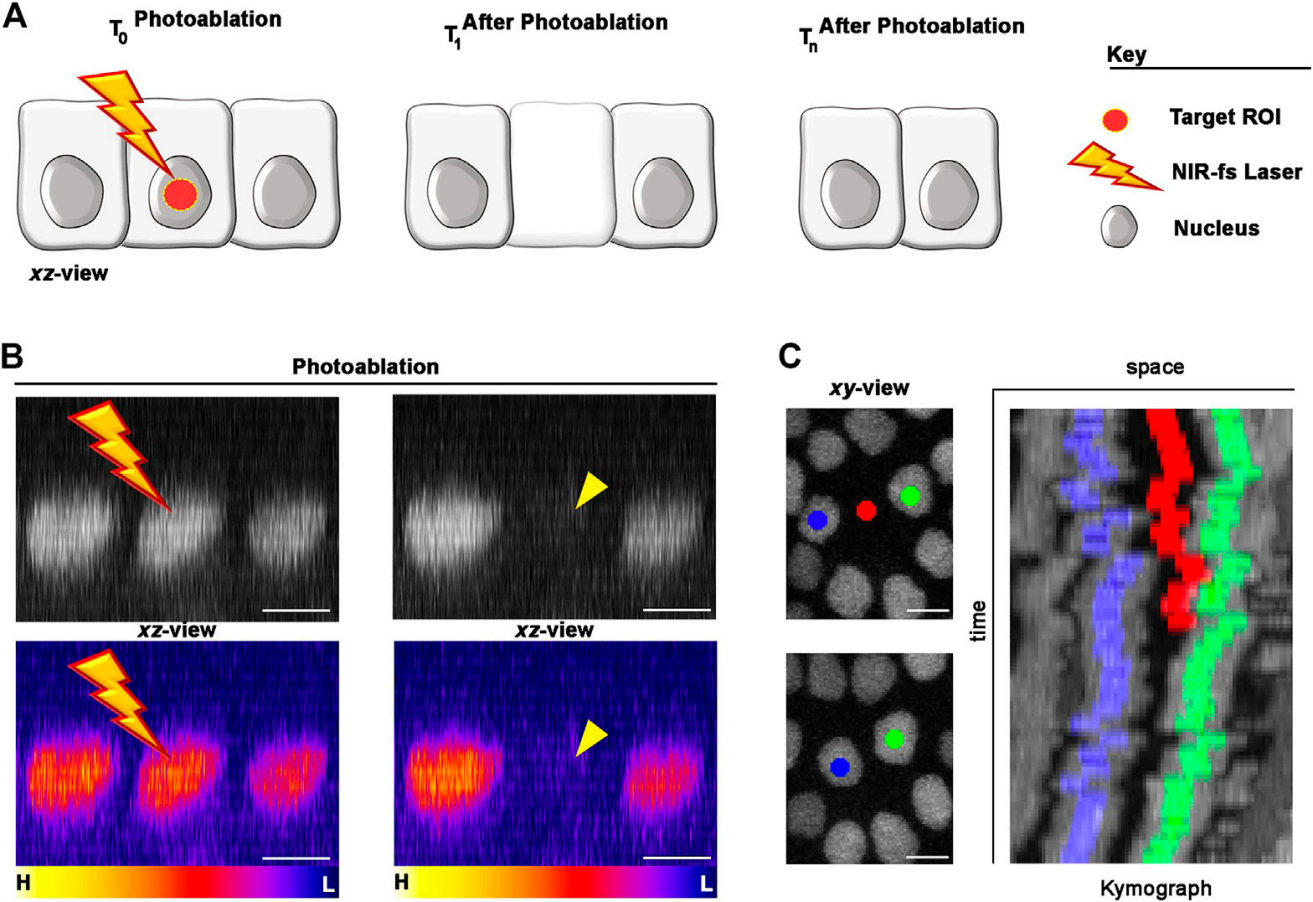
Photoablation of single cells: targeting precision *in vivo*. **(A)** Diagram of the photoablation process. The targeted ROI is in red (left) and the cellular responses after photoablation are illustrated (canter and right). **(B)** An example of *in vivo* photoablation of a single epidermal cell in the *Drosophila* epidermis at 40 hAPF. Top: the nuclei of three neighbouring epidermal cells (side view), marked by the expression of *Diap1-GFP* (grey), are shown before (left) and 10 s after (right) photoablation. Bottom: Pseudo-coloured image for fluorescent intensities (yellow-orange, high; purple-blue, low) showing a drop of signal intensity in the targeted cell after photoablation but not in the neighbouring cells. Photoablation was performed with the NIR-fs pulsed laser tuned at 780 nm, 70% power, 1.02 µs dwell time and 1 iteration within a circular ROI of 25 pixels in diameter (about 1.7 µm). See Supplementary Video S1. **(C)** Images for a time lapse showing the position of the photo-ablated cell (red) and its replacement by neighbouring cells (blue and green). Kymograph showing dynamic trajectory of the photo-ablated cell (red), which delaminates from the epidermis and neighbouring cells (blue and green) that seal the gap overtime. Scale bars: 5 µm. See Supplementary Video S2. This experiment was repeated 8 times with equivalent results (*n* = 8 pupae).

To perform single cell photoablation, we focused the NIR-fs pulsed laser at the centre of nucleus of the targeted cell (Figure 3A), designing a circular ROI using the software of the microscope (see Materials and Equipment). We recommend drawing circular ROIs with diameter no more than half of the nuclear diameter. For example, for a cell nucleus of 5 μm diameter, we recommend designing a ROI of no more than 2 μm of diameter. This strategy helps limiting the extend of thermo-mechanical damage, which expands after laser pulse delivery (Vogel et al., 2005; Vogel et al., 2008; Tsai et al., 2009), to the targeted cell.

Nuclear markers are particularly useful in photoablation experiments, as nuclei between adjoining cells are appreciably separated. In the example provided in Figures 3B,C we marked the nuclei of the epidermal cells with the expression of a *Death-associated inhibitor of apoptosis 1*—GFP reporter (*Diap1-GFP*). To precisely deliver high NIR photon density at the centre of the targeted cell volume, we used high NA objective lens (1.4 NA) and high laser power. High NA objectives reduce out-of-focus damage neighbouring cells, while high laser power induce thermo-mechanical damage at the target site. This ensures that the region of highest energy density corresponds to the centre of the cell nucleus (Figures 3A,B), where the photodamage induced by the laser will be confined. Thus, the ROI size and focal plane are important parameters that needs to be adjusted according to the nuclear size/shape of the target cell.

We achieved the best results tuning the NIR-fs pulsed laser at 780 nm but, as laser ablation is mediated through thermo-mechanical damage, the use of a specific wavelength is not critical. Instead, other parameters including laser power, pixel dwell and number of iterations, are all crucial for successful laser ablation within the tissue. We set the NIR-fs laser power at 70% and used a pixel dwell between of 1–2 μs. Together, these parameters allow an efficient photoablation in epidermal cells (Figure 3B; Supplementary Video S1) with a single iteration. We recommend performing a time-lapse imaging experiment post-ablation to verify the extrusion of the targeted cell (Figure 3C; Supplementary Video S2). Taken together, these data show that our system is well suited for photoablation at single cell resolution. The photodamage is here confined to the targeted cell, leaving surrounding cells unaltered. In the next section, we show how we applied this methodology for studying cellular behaviours within tactile bristles or surrounding epidermis in the differentiating PNS.

**FIGURE 4:**
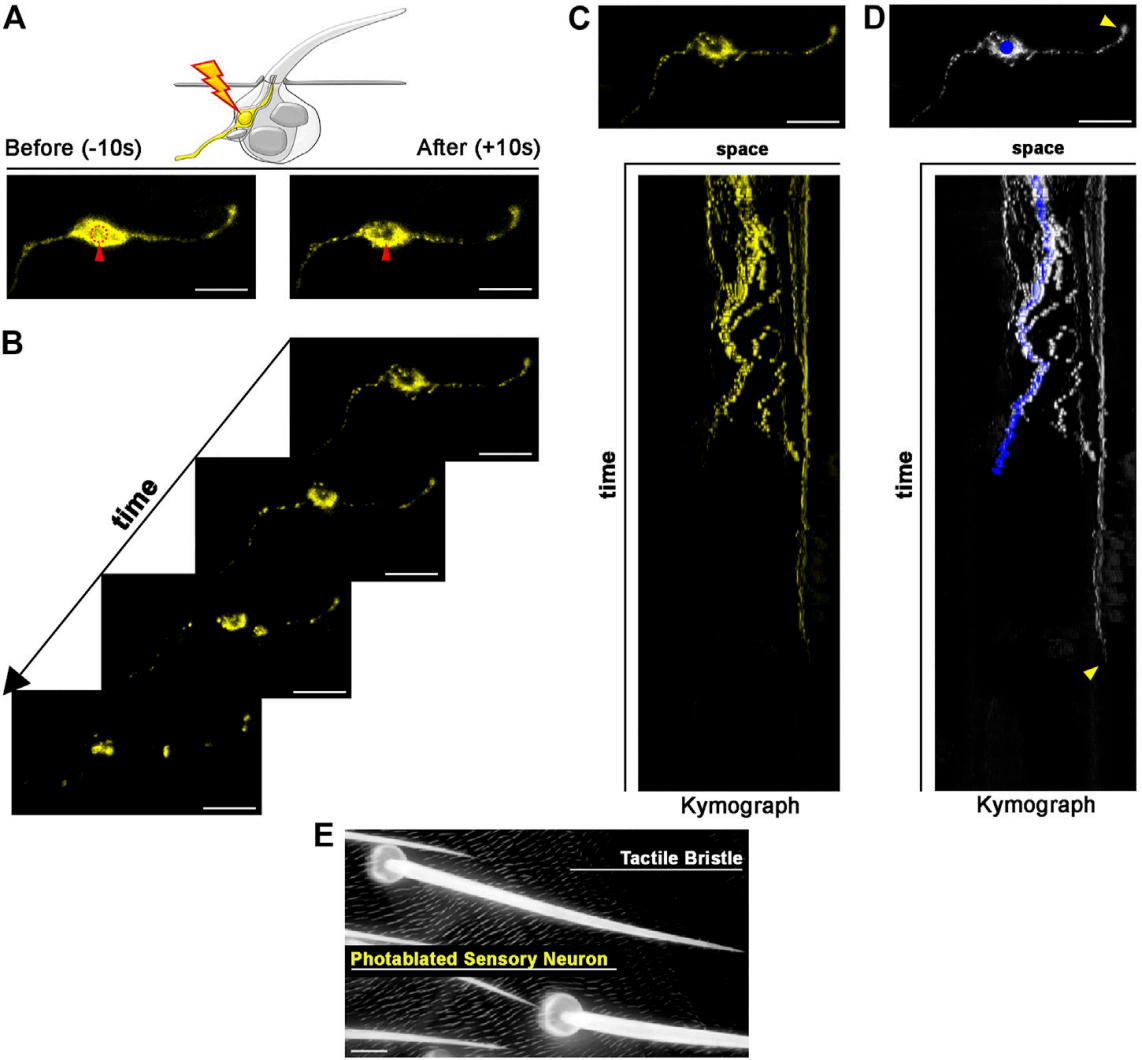
Photoablation of peripheral mechanosensory neurons. Images showing ablation of the sensory neuron of the tactile bristle. The shape of the neuron is marked by the expression of *UAS-mCD8::GFP* (yellow) under the control of *elav-GAL4*. Note the immediate sign of damage after ablation. Photoablation was performed with the NIR-fs pulsed laser tuned at 780 nm, 70% power, 2.04 µs dwell time and two iterations within a circular ROI of 20 pixels in diameter (about 1.4 µm). See Supplementary Video S3. **(B)** Through time, the ablated neuron is undergoing cell death. **(C,D)** Kymographs showing dynamic trajectory of the sensory neuron cell body and dendrite after photoablation. Note that the distal dendrite of the neuron (arrowhead) disappears well after the fragmentation of the neuron cell body (blue dot) and distal axon. **(E)** Images of the adult cuticle and associated tactile organs after photoablation of the neuron during bristle differentiation. Scale bars: 5 µm **(A–D)** and 10 µm **(E)**. See Supplementary Video S4. This experiment was repeated 4 times with equivalent results (*n* = 4 pupae).

Notes:

1) Proper alignment in the z-plane of the NIR-fs pulsed laser with the detection beam path is crucial for successful photoablation (or imaging in MP mode). Most microscope manufacture have integrated motorized periscopes and tool tabs to perform this operation.
2) Identifying the appropriate ROI size and laser power requires some experimentation, which should be undertaken before performing the photoablation experiment.
3) After identifying the appropriate laser power, that is, sufficient to ablate a given ROI with a single scan, these settings will give reproducible results if applied at equivalent depths within the same tissue.
4) ROIs positioned deep into a tissue will generally require higher laser power to achieve the same effect.
5) If photoablation is unsuccessful, we recommend optimizing the parameters described in this protocol one at a time, starting with increasing the number of iterations or the laser dwell time.
6) Insufficient laser power will not eliminate the targeted cell and will only cause photobleaching of the fluorescence signal.
7) Laser power should be increased gradually to find the minimum level required for successful ablation. Too high laser power, increased dwell time or excess number of iterations will damage not only the target cell but also the surrounding area.
8) The cell membrane can also be used to define the target region and run a successful single cell photoablation (see Figure 4).

## Results

### Photoablation of peripheral mechanosensory neurons

Each tactile bristle of the *Drosophila* PNS is innervated by a single bipolar sensory neuron (Hartenstein and Posakony, 1989; Keil, 1997). The dendrite is fully encapsulated by the tactile bristle cells, while the sensory neuron cell body and axon reside basally to the epidermal cuticle, suggesting that dendrites and axons could behave differently after photoablation of the cell body. To test this hypothesis, we highlighted neuronal morphology by expressing a membrane-tagged GFP (*UAS-CD8::GFP*) under the control of the neuronal driver *embryonic lethal abnormal vision* (*elav*)-*GAL4* (Figure 4). We then performed targeted photoablation of the neuron cell body (Figure 4A; Supplementary Video S3) and subsequent live imaging to monitor cell behaviour over time (Figure 4B; Supplementary Video S4). The induced photodamage was visible by reduced fluorescent expression of GFP at the site of ablation and fragmentation of the axon, cell body and dendrite (Figures 4B,C), indicating cell death in response to photodamage. Additionally, these experiments further revealed that the dendrite tip persists attached to the bristle, as it retracts after the death of the neuron (Figure 4D). This observation suggests bristle cells protect the distal-most part of dendrite. It should be noted that, in this experiment, we performed successful photoablation using only the membrane marker to direct photodamage to the neuron. In conclusion, the photoablation strategy described here efficiently eliminate sensory neurons in the *Drosophila* PNS.

### Morphological modularity of bristle cells in response to photoablation

**FIGURE 5:**
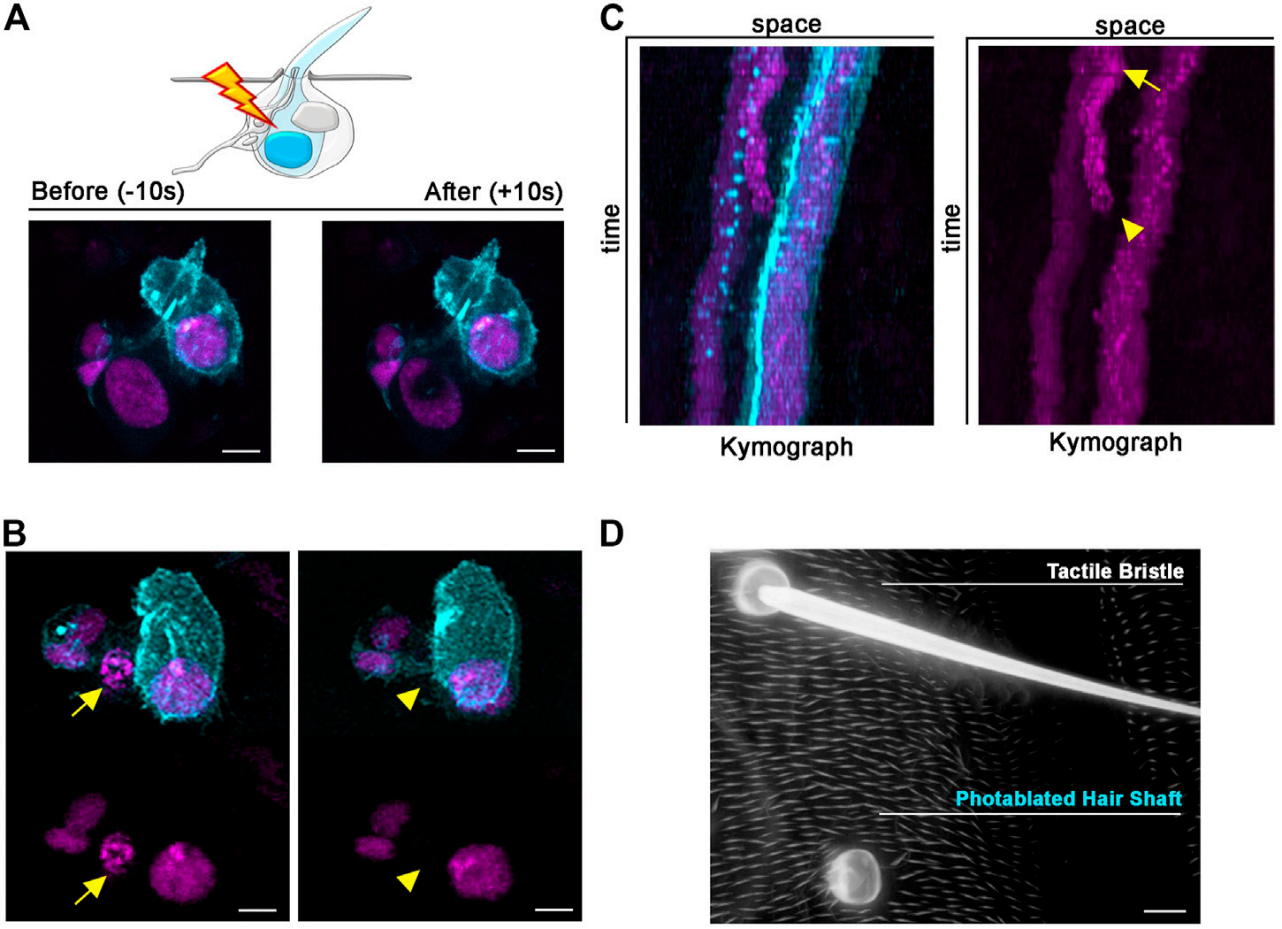
Long-term effects of Shaft cell ablation into adult bristle morphology. **(A)** Diagram and images showing ablation of the differentiating Shaft cell of the tactile bristle. The nuclei and membrane of the bristle cells are marked by simultaneous expression of *UAS-RFP. nls* (magenta) and *UAS-Actin::GFP* (cyan) under control of *neur-GAL4*. Note the immediate sign of damage after ablation. Photoablation was performed with the NIR-fs pulsed laser tuned at 780 nm, 70% power, 2.04 µs dwell time and 1 iteration within a circular ROI 35 pixels in diameter (about 2.4 µm). **(B)** Images from a time lapse showing nuclear condensation (left, arrow) and disappearance (right, arrowhead) of the Shaft cell after photoablation (left). **(C)** Kymographs showing dynamic trajectory of bristle cells after photoablation. The targeted Shaft cell (arrow) delaminates at subsequent time points (arrowhead), while the other cells of the bristles remain unaffected. **(D)** Images of the adult cuticle and associated tactile organs after photoablation of the Shaft cell during bristle differentiation. Note that the bristle socket has developed normally in absence of the Shaft cell. Scale bars: 5 µm **(A,B)** and 10 µm **(C)**. See Supplementary Video S5, S6. This experiment was repeated 8 times with equivalent results (*n* = 6 pupae).

All bristle cells originate from a unique precursor cell after four rounds of asymmetric divisions that occur during the pupal stages (Hartenstein and Posakony, 1989; Gho et al., 1996). Upon completion of the lineage, bristle cells enter a postmitotic stage in which each cell executes a complex program of differentiation, to build the adult mechanosensory bristle. For example, the Shaft cell produces the hair shaft, which is deflected in response mechanical stimuli, and the Socket cell builds the cup-like cuticular structure that forms the base of the hair shaft, while contributing to the ionic microenvironment of the tactile organ (Barolo et al., 2000; Jarman, 2002). The Socket and Shaft are sister cells in the SOP lineage, making their individual manipulation particularly challenging. A common outcome of many genetic manipulations at the time of Socket/Shaft cell fate decision is cell fate transformation, leading to adult tactile bristles with two socket-like structures or two hair shaft-like structures (Schweisguth and Posakony, 1994; Barolo et al., 2000). Genetic manipulations that lead to a bristle with one Shaft but no Socket structure and *vice versa*, are rare or appear at low penetrance, limiting experimental reproducibility. Even with these tools, temporal control of cell elimination is very challenging, in contrast to our method. Moreover, combining those mutant backgrounds with fluorescent reporters for live imaging is often challenging. Photoablation would therefore provide a unique opportunity to elucidate whether a morphologically normal hair socket or hair shaft structure can be formed in complete absence of the corresponding sister cell. Furthermore, the close association of the Shaft and Socket cells during development provides an ideal testing ground to ask if photo-ablation of 1 cell leads to damage in its neighbour. In the example provided in Figure 5, we apply our protocol to photo-ablate the Shaft cell only. We used the bristle-specific driver *neur-GAL4* to drive expression of a nuclear marker tagged with RFP (*UAS-H2B::RFP*) and membrane localized GFP (*UAS-Actin::GFP*). In this way, all bristle nuclei and cellular structures are visible during and after the experiment (Figure 5A; Supplementary Video S5). *In vivo* imaging of the differentiating tactile bristle showed that the targeted Shaft cell undergoes cell death, leaving all others bristle cells unaffected (Figures 5B,C; Supplementary Video S6). Furthermore, we inspected the impact of our ablations to the adult organ. We found that the Socket cell developed the cuticular structure typical of the hair base in the absence of the Shaft cell (Figure 5D). This experiment revealed that the presence of the Socket cell alone is sufficient to produce a fully differentiated hair socket in the adult fly.

**FIGURE 6:**
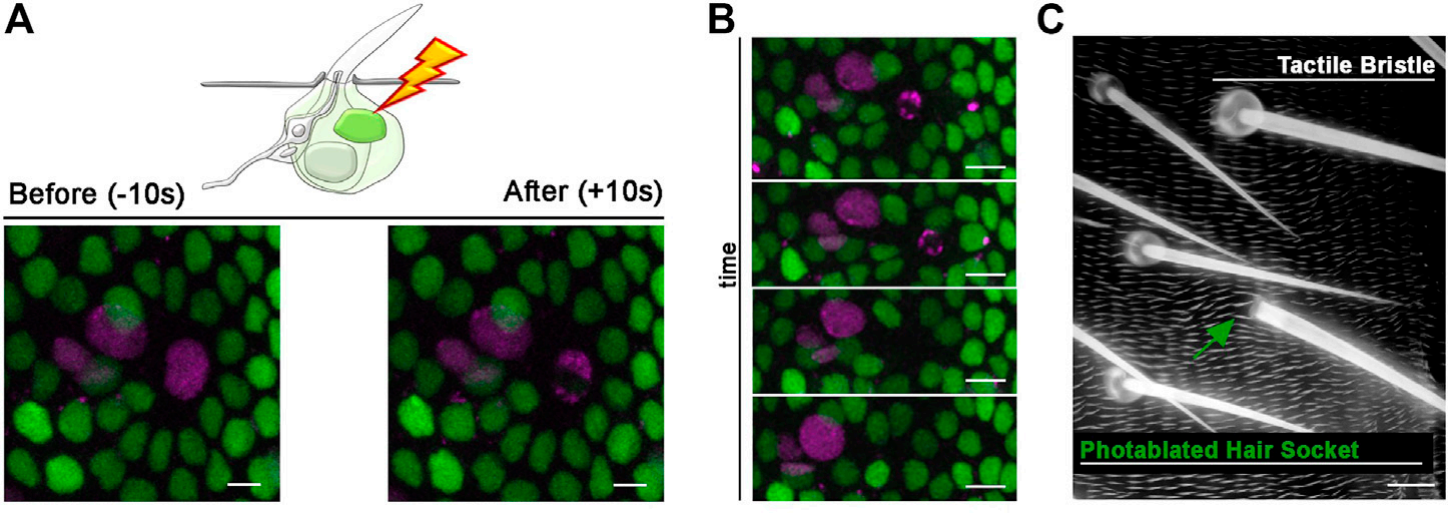
Photoablation of the Socket cell and effect on the adult tactile bristle. **(A)** Diagram and images showing ablation of the differentiating Socket cell of the tactile bristle. The nuclei of the bristle cells are marked by the expression of *UAS-RFP. nls* (magenta) under the control of *neur-GAL4*. All nuclei are marked by the expression of *Diap1-GFP* (green). Note the immediate sign of nuclear damage after ablation. Photoablation was performed with the NIR-fs pulsed laser tuned at 780 nm, 70% power, 2.04 µs dwell time and 1 iteration within a circular ROI of 30 pixels in diameter (about 2 µm). See Supplementary Video S7. **(B)** Through time, the ablated cell is extruded from the epidermis and the bristle undergoes differentiation without a Socket cell. **(C)** Images of the adult cuticle and associated sensory organs after photoablation of the Socket cell during bristle differentiation. Note that the hair shaft has developed normally in absence of the Socket cell. Scale bars: 5 µm **(A,B)** and 10 µm **(C)**. See Supplementary Video S8. This experiment was repeated 8 times with equivalent results (*n* = 6 pupae).

We next used our protocol to photo-ablate the Socket cell. For this experiment, we marked bristle nuclei (*neur-GAL4, UAS-H2B::RFP*) and the surrounding epidermal cells with the expression of the *Diap1-GFP* nuclear reporter (Figure 6). After photoablation of the Socket cell (Figure 6A; Supplementary Video S7), we performed *in vivo* imaging to follow the response of cells surrounding the photodamaged Socket (Figure 6B; Supplementary Video S8). We observed that, through time, epidermal cells rearrange their positions to seal the space previously occupied by the socket cell, which underwent cell death, while remaining bristle cells continue differentiation (Figure 6B). We then evaluated the impact of Socket cell loss on the adult tactile organ by visualizing the adult epidermal cuticle. We found that the Shaft cell correctly secreted the hair shaft structure and the epidermis surrounding the “Socket-less” bristle appears normal (Figure 6C). This experiment revealed that the presence of the Shaft cell alone is sufficient to produce a fully differentiated hair shaft in the adult fly.

Taken together these experiments show that the Socket and the Shaft cells of the bristle, are relatively autonomous with respect to each other in their differentiation, highlighting a morphological modularity in the making of the adult tactile organ. Furthermore, these data provide a stringent test showing that ablation of one sister in a pair of closely associated cells does not impair the terminal differentiation of the other. Thus, our photoablation strategy is an effective tool to selectively ablate cells within the bristle and will help gaining insights into tactile bristle differentiation.

### Intercellular rearrangements after epidermal photoablation

**FIGURE 7:**
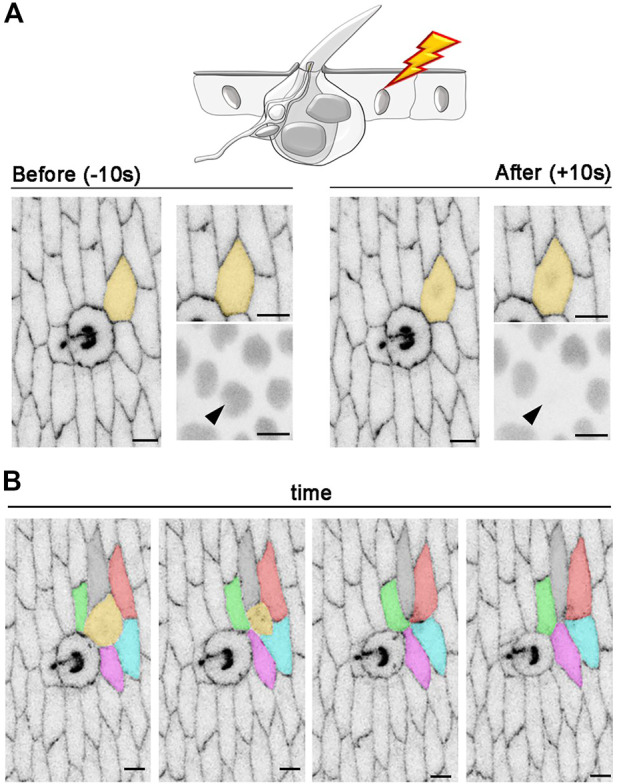
Intercellular rearrangements after photoablation of a single epidermal cell. **(A)** An example of *in vivo* photoablation of a single epidermal cell in contact with a tactile bristle at 55 hAPF. Cell outlines are marked with *E-Cad::GFP* (grey) and nuclei are marked by the expression of *Ubi-RFP. nls* (grey). The targeted cell is highlighted in orange and arrowhead. Photoablation was performed with the NIR-fs pulsed laser tuned at 780 nm, 70% power, 1.02 µs dwell time and 1 iteration within a circular ROI of 25 pixels in diameter (about 1.7 µm). See Supplementary Video S9. **(B)** Images from a time-lapse imaging showing extrusion and intercellular dynamics of a photo-ablated epidermal cell in contact with a neighbouring bristle. Note that, through time, the targeted cell (orange) is extruded from the tissue and new cell contacts are established between the remaining cells. Scale bars: 5 µm. See Supplementary Video S10. This experiment was repeated 4 times with equivalent results (*n* = 4 pupae).

To further test the spatial precision of our photoablation strategy, we selectively targeted single epidermal cells surrounding the tactile bristle. In the experiment presented in Figure 7, we used photoablation to target a single epidermal cell in contact with the Socket cell. To visualize both epidermal and bristle cells, we used *E-Cadherin::GFP* (*E-Cad::GFP*), which localizes to the epithelial adherens junctions, together with the ubiquitous expression of nuclear RFP (*Ubi-RFP.nls*) (Figure 7A; Supplementary Video S9). After photoablation, the target cell showed reduction in surface area and subsequent extrusion, while surrounding cells rearranged their connections to close the gap (Figure 7B; Supplementary Video S10). This experiment confirms that our method allows localized damage to the targeted cell. Additionally, it makes the targeting of individual epidermal cells feasible, which would otherwise be very labour-intensive to achieve genetically.

## Conclusion

In this study, we described how to efficiently employ a NIR-fs pulsed laser for precise targeting and subsequent elimination of the desired cell type, at the desired time. Using the photoablation method we have implemented, we showed how cell damage can be precisely and reproducibly confined to single cells, while avoiding neighbouring cells. A NIR-MP laser was used to achieve single cell ablation with high precision in a high heterogeneous cellular context such as the *Drosophila* PNS at pupal stages. Diverse cellular behaviours including cell extrusion, cell rearrangements and cell shape changes can be monitored *in vivo* immediately after photodamage, and for several hours using high resolution confocal microscopy. This methodology can circumvent many of the technical challenges associated with the lack of genetic tools, complex genetic backgrounds or the difficulty associated to their precise temporal control. Together with the ensuing spatial and temporal flexibility inherent to laser ablations, the application of our protocol ensures localized damage to the targeted cell. Overall, this protocol provides a simple and versatile method to target individual cells within heterogenous tissues with high fidelity. We anticipate that this methodology, together with the use of genetic tools and further cell biological studies, will help deciphering the interaction between neuronal and non-neuronal cells in the complex landscape of the nervous system.

## Data Availability

The original contributions presented in the study are included in the article/Supplementary Material, further inquiries can be directed to the corresponding author.

## Supplementary material

Supplementary Video S1 Photoablation at single cell resolution. Photoablation of a single epidermal cell at 40 hAPF using the NIR-fs laser line tuned at 780 nm and 70% power. A transverse view of the epithelium (*z*-dimension) is shown. The nuclei of the epidermal cells are labelled by the expression of *Diap1-GFP* and displayed in grey scale (left) or pseudo-coloured (right) for signal intensities (yellow-orange, high; purple-blue, low). Time interval is 10 s. Scale bar is 5 μm. See also Figure 3.

Supplementary Video S2 Cellular dynamics after single cell photoablation. Time-lapse imaging of epidermal cells after photoablation of a single epidermal cell at 40 hAPF. The photo-ablated cell is marked with a red dot. The left and right cell neighbours are marked with a blue and a green dot, respectively. Nuclei are labelled by the expression of *Diap1-GFP*. Scale bar is 5 μm. Time interval is 5 min. See also Figure 3.

Supplementary Video S3 Photoablation of the mechanosensory neuron of a tactile bristle. Photoablation of the mechanosensory neuron innervating a differentiating tactile bristle at 60 hAPF using the NIR-fs pulsed laser tuned at 780 nm and 70% power. The mechanosensory neuron is marked by the expression of membrane-localized GFP (*elav-GAL4, UAS-mCD8::GFP*; yellow). Scale bar is 5 μm. Time interval is 10 s. See also Figure 4.

Supplementary Video S4 Cellular dynamics after photoablation of the mechanosensory neuron. Time-lapse imaging of a sensory neuron after photoablation. The shape of the mechanosensory neuron is marked by the expression of membrane-localized GFP (*elav-GAL4, UAS-mCD8::GFP*; yellow). Scale bar is 5 μm. Time interval is 1 min. See also Figure 4.

Supplementary Video S5 Photoablation of the Shaft cell of the tactile bristle. Photoablation of the Shaft cell at 40 hAPF using the NIR-fs pulsed laser tuned at 780 nm and 70% power. The cells of the bristles are marked by simultaneous expression of membrane-localized GFP (cyan) and nuclear RFP (magenta) (*neur-GAL4, UAS-Actin::GFP, UAS-H2B::RFP*). Scale bar is 5 μm. Time interval is 10 s. See also Figure 5.

Supplementary Video S6 Cellular dynamics after photoablation of the Shaft cell of the tactile bristle. Time-lapse imaging of a tactile bristle after photoablation of the Shaft cell at 40 hAPF. The cells of the bristles are marked by simultaneous expression of membrane-localized GFP (cyan) and nuclear RFP (magenta) (*neur-GAL4, UAS-Actin::GFP, UAS-H2B::RFP*). Scale bar is 5 μm. Time interval is 5 min. See also Figure 5.

Supplementary Video S7 Photoablation of the Socket cell of the tactile bristle. Photoablation of the Socket cell at 40 hAPF using the NIR-fs pulsed laser tuned at 780 nm and 70% power. The cells of the bristles are marked by expression of nuclear RFP (*neur-GAL4, UAS-H2B-RFP*; magenta). The epidermal cells are marked by the expression *Diap1-GFP* (green). Scale bar is 5 μm. Time interval is 10 s. See also Figure 6.

Supplementary Video S8 Cellular dynamics after photoablation of the Socket cell of the tactile bristle. Time-lapse imaging of a tactile bristle after photoablation of the Socket cell at 40 hAPF. The cells of the bristles are marked by expression of nuclear RFP (*neur-GAL4, UAS-H2B.RFP*; magenta). The epidermal cells are marked by the expression *Diap1-GFP* (green) to visualize cell rearrangements after photoablation of the Socket cell of the bristle. Scale bar is 5 μm. Time interval is 5 min. See also Figure 6.

Supplementary Video S9 Photoablation of a single epidermal cell surrounding the tactile bristle. Photoablation of an epidermal cell in contact with the Socket cell at 55 hAPF using the NIR-fs pulsed laser tuned at 780 nm and 70% power. The cell outlines are marked by the expression of the junctional marker *E-Cad::GFP*. Scale bar is 5 μm. Time interval is 10 s. See also Figure 7.

Supplementary Video S10 Intercellular rearrangements between tactile bristles and epidermis after photoablation. Time-lapse imaging showing the cellular rearrangements after the ablation of a single epidermal cell in contact with the Socket cell of the bristle at 55 hAPF. The cell outlines are marked by the expression of the junctional marker *E-Cad::GFP*. Scale bar is 5 μm. Scale bar is 5 μm. Time interval is 2 min. See also Figure 7.
